# Cociente ácido úrico/linfocitos como mediador entre comorbilidad y riesgo de caídas en adultos mayores

**DOI:** 10.1016/j.aprim.2026.103611

**Published:** 2026-09-22

**Authors:** Abir Alsalahi Taha, Alejandro Sosa Jiménez, Juan Abreu González

**Affiliations:** a Gerencia Atención Primaria, Tenerife, España; b Unidad de investigación, Hospital Universitario de Canariasm, La Laguna, Santa Cruz de Tenerife, España; c Departamento de Neumología, Hospital Universitario de Canarias, La Laguna, La Laguna, Santa Cruz de Tenerife, España; d Universidad de La Laguna, La Laguna, Santa Cruz de Tenerife, España

**Keywords:** Fragilidad, Accidentes por caídas, Ácido úrico, Linfocitos, Comorbilidad, Atención primaria de salud, Frailty, Accidental falls, Uric acid, Lymphocytes, Comorbidity, Primary health care

## Abstract

**Objetivo:**

Cuantificar la proporción de la asociación entre comorbilidad y riesgo de caídas mediada por el cociente ácido úrico/linfocitos (UALR) en personas mayores, y evaluar su invarianza por sexo.

**Diseño:**

Estudio observacional de cohorte longitudinal retrospectiva, con seguimiento de 5 años.

**Emplazamiento:**

Atención primaria.

**Participantes:**

310 personas no institucionalizadas de 70-85 años (52,6% mujeres) en seguimiento activo.

**Mediciones principales:**

Índice de comorbilidad de Charlson (CCI); ácido úrico/linfocitos (UALR) basal y su cambio; riesgo de caídas incidente (índice de Downton ≥ 3). La mediación se estimó con ecuaciones estructurales (SEM-WLSMV) y bootstrap BCa, con réplica mediante PROCESS (SPSS); la invarianza por sexo con la prueba Δχ^2^ de Satorra-Bentler.

**Resultados:**

El efecto indirecto del UALR fue significativo (a × b = 0,040; IC del 95% 0,007-0,072; p = 0,016), con una proporción mediada del 33,8% (p = 0,054). La asociación UALR → riesgo de caídas (B = 0,402; p< 0,001; OR = 1,49) fue unas 4 veces mayor que CCI → UALR (B = 0,099; p = 0,003). El efecto directo fue limítrofe (c’ = 0,078; p = 0,060). No se detectaron diferencias por sexo (Δχ^2^ = 1,133; p = 0,769), con potencia limitada.

**Conclusiones:**

El UALR media una parte sustancial de la asociación entre la comorbilidad y el riesgo de caídas incidente, con el que se asocia con más fuerza que la propia comorbilidad. Obtenible de una analítica rutinaria, podría ser útil para la estratificación del riesgo en atención primaria, pendiente de validación en cohortes prospectivas independientes.

## Abstract (en)

**Objective:**

To quantify the proportion of the association between comorbidity and fall risk mediated by the uric acid-to-lymphocyte ratio (UALR) in older adults, and to assess its invariance across sex.

**Design:**

Observational retrospective longitudinal cohort study, with 5-year follow-up.

**Participants:**

310 community-dwelling adults aged 70-85 years (52.6% women) under active follow-up.

**Main measurements:**

Charlson Comorbidity Index (CCI); baseline uric acid/lymphocytes (UALR) and its change; incident high fall risk (Downton fall risk index ≥3). Mediation was estimated using structural equation modeling (SEM-WLSMV) with bias-corrected and accelerated (BCa) bootstrap and replicated using the PROCESS macro for SPSS; sex invariance with the Satorra-Bentler Δχ^2^ test.

**Results:**

The indirect effect of UALR was significant (a×b = 0,040; 95%CI 0,007-0,072; p = 0,016), with a proportion mediated of 33.8% (p = 0,054). The UALR → fall-risk association (B = 0,402; *P*<0,001; OR = 1.49) was about 4 times larger than CCI → UALR (B = 0.099; p = 0.003). The direct effect was borderline (c’ = 0,078; p = 0,060). No sex differences were detected (Δχ^2^ = 1.133; p = 0,769), with limited power.

**Conclusions:**

UALR mediates a substantial part of the comorbidity–incident high fall-risk association and is more strongly associated with falls than comorbidity itself. Obtainable from routine blood tests, it could be useful for risk stratification in primary care, pending validation in independent prospective cohorts.

## Introducción

La fragilidad es un síndrome geriátrico multidimensional, caracterizado por el declive de la reserva fisiológica y una mayor vulnerabilidad, que conlleva un riesgo aumentado de caídas, hospitalización, institucionalización y muerte^1^, ^2^. Su prevalencia en la comunidad en mayores de 65 años es del 10,7%^3^ y su detección precoz es prioritaria, ya que las intervenciones multicomponente pueden frenar su progresión^4^. Las caídas constituyen uno de sus eventos adversos más relevantes, y la fragilidad es un predictor independiente bien establecido del riesgo de caídas^5^, ^6^.

El índice de Downton es una herramienta pragmática y ampliamente utilizada para evaluar el riesgo de caídas en mayores^7^. Conviene precisar que mide riesgo de caídas y no fragilidad; no obstante, sus ítems más discriminativos —alteración de la marcha y deterioro cognitivo— solapan con dominios nucleares del fenotipo de Fried, y un riesgo de caídas elevado se ha asociado de forma independiente con la fragilidad (OR = 3,42; IC del 95%: 1,22-9,53)^8^. En este trabajo, el Downton ≥ 3 se utiliza como indicador de vulnerabilidad funcional asociada a caídas, sin asumir equivalencia con instrumentos validados de fragilidad.

La comorbilidad, medida con el índice de Charlson (CCI), se asocia de forma consistente con la fragilidad^9^, aunque sus mecanismos no se conocen por completo. La inflamación crónica de bajo grado *(inflammaging)* se ha propuesto como mecanismo clave^10^, si bien los biomarcadores unidimensionales muestran una capacidad predictiva longitudinal limitada: en el metaanálisis de Soysal et al.^11^, la proteína C reactiva (PCR) y la interleucina 6 (IL-6) estaban elevadas en sujetos frágiles pero no predijeron la fragilidad incidente, y Samson et al.^12^ describieron una precisión del 10-12% con biomarcadores combinados a 20 años, frente a la asociación más fuerte del estrés oxidativo con la fragilidad^13^. Ello refuerza la necesidad de índices que integren varias dimensiones fisiopatológicas.

El UALR representa un enfoque integrador: combina la carga oxidativa del catabolismo de las purinas^14^, ^15^ con la reserva inmunitaria reflejada en el recuento linfocitario^16^, aproximando la desregulación multisistémica de la fragilidad^17^. Propuesto en contextos clínicos agudos^18^, ^19^, su aplicabilidad en población comunitaria está poco establecida y no se ha examinado su relación con el riesgo de caídas ni su contribución mediadora en la asociación comorbilidad-caídas. Tampoco se han explorado diferencias por sexo, pese al conocido dimorfismo del ácido úrico^20^. El objetivo de este estudio fue cuantificar la proporción de la asociación CCI → riesgo de caídas mediada por el UALR y evaluar su invarianza por sexo.

## Material y métodos

### Diseño, población y selección

Análisis secundario de una cohorte retrospectiva de un centro de Atención Primaria, constituida inicialmente con otra finalidad, con datos extraídos de la historia clínica electrónica. Se evaluó la elegibilidad de los pacientes de 70-85 años registrados en el centro entre 2019 y 2024. Los criterios de exclusión fueron: datos de laboratorio incompletos, institucionalización, hospitalización reciente (< 3 meses), presencia de síntomas agudos, enfermedad terminal y ausencia de seguimiento activo. La cohorte original se constituyó para el estudio de biomarcadores hematológicos, y su reutilización para esta pregunta se planificó a posteriori. La identificación de los pacientes fue consecutiva a partir del registro del centro, sin muestreo aleatorio. De 1.130 pacientes evaluados se excluyeron 820, resultando una muestra final de 310; por tratarse de una extracción retrospectiva, no fue posible recuperar el número de exclusiones atribuible a cada criterio de forma individual. El esquema del estudio se presenta en la figura 1.

**Figura 1:**
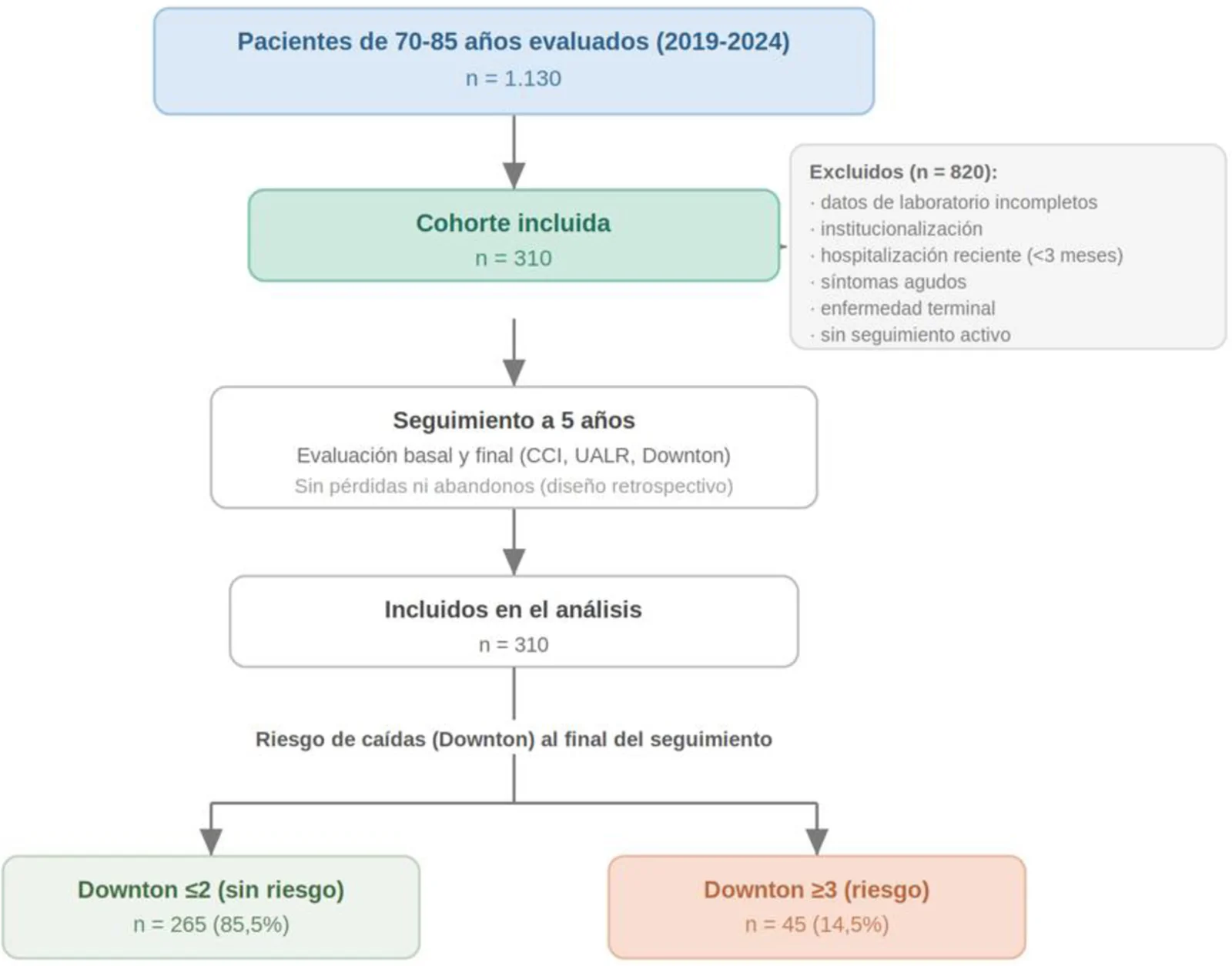
Esquema del estudio. De 1.130 personas de 70-85 años evaluadas para elegibilidad (2019-2024) se excluyeron 820 (datos de laboratorio incompletos, institucionalización, hospitalización reciente < 3 meses, síntomas agudos, enfermedad terminal o ausencia de seguimiento activo), resultando una cohorte de 310 participantes. Por el diseño retrospectivo no hubo pérdidas durante el seguimiento. Ningún participante presentaba riesgo elevado de caídas en la basal; los 45 casos al final del seguimiento son incidentes.

### Variables

Variable independiente. El CCI se calculó según Charlson et al.^21^, con 19 categorías ponderadas de comorbilidad.

Mediador. El UALR se calculó como el cociente entre ácido úrico sérico (mg/dl) y recuento absoluto de linfocitos (× 10^9^/l), a partir de sangre venosa en ayunas en ambos momentos; se analizaron su valor basal y su cambio longitudinal (ΔUALR).

Desenlace. El desenlace fue el riesgo de caídas, definido como una puntuación del índice de Downton ^22^ ≥3. Se clasificó a los participantes en Downton ≤ 2 frente a ≥ 3. El índice constaba registrado en la historia clínica electrónica tanto en la evaluación basal (2019) como en la final (2024); los valores se recuperaron directamente de la historia, sin recálculo ni imputación. En la basal, ningún participante presentaba una puntuación ≥ 3 (distribución: 0, n = 235; 1, n = 39; 2, n = 36), por lo que los 45 casos registrados al final del seguimiento (14,5%; 45/310) constituyen riesgo de caídas incidente.

Covariables. Edad, sexo, índice de masa corporal (IMC), uso de diuréticos tiazídicos y filtrado glomerular estimado (FGe, CKD-EPI), incluido por su correlación inversa moderada con el UALR (ρ de Spearman = −0,30; p < 0,001).

### Análisis estadístico

La mediación se estimó mediante ecuaciones estructurales con estimador WLSMV, apropiado para desenlaces categóricos/ordinales^23^, especificando la vía a (CCI → UALR), la vía b (UALR → riesgo de caídas, ajustada por CCI) y el efecto directo c’. El efecto indirecto (a × b) se contrastó con el test de Sobel y bootstrap BCa (5.000 remuestreos)^24^; la proporción mediada como a × b/(a × b + c’). La invarianza por sexo se evaluó con SEM multigrupo y la prueba Δχ^2^ escalado de Satorra-Bentler^25^. Como análisis de sensibilidad, la mediación (X → M → Y) se replicó como mediación simple con desenlace logístico mediante la macro PROCESS (modelo 4) de Hayes^26^, con bootstrap de 5.000 remuestreos, y de forma concordante en R; se exploraron 4 modelos de mediación serial con biomarcadores distales (cocientes plaquetas/linfocitos [ΔPLR], neutrófilos/linfocitos [ΔNLR], Δalbúmina y Δácido úrico); la evolución temporal del UALR se analizó mediante un modelo lineal mixto con intercepto aleatorio por sujeto. Los índices de ajuste se interpretaron según Hu y Bentler^27^. Los análisis se realizaron con R (paquete lavaan para los modelos de ecuaciones estructurales con estimador WLSMV y el bootstrap BCa; modelos de regresión, modelos lineales mixtos y comparaciones descriptivas —t de Student, χ^2^ y U de Mann-Whitney—) y con IBM SPSS Statistics (macro PROCESS de Hayes para el análisis de sensibilidad de mediación). Los contrastes fueron bilaterales (p < 0,05) y la comunicación siguió la guía STROBE.

Consideraciones sobre el tamaño muestral. Con 45 casos, el número de eventos es reducido en relación con la complejidad de los modelos: el cociente de eventos por parámetro del modelo confirmatorio se mantuvo próximo a 10, mientras que los modelos multigrupo quedaron por debajo de ese umbral. Por ello, los modelos confirmatorios se especificaron de forma parsimoniosa y el análisis multigrupo y de mediación serial se consideran exploratorios, con posible sobreajuste. La identificación del efecto de mediación descansa en el supuesto no verificable de ausencia de confusión no medida (ignorabilidad secuencial); pese al ajuste por edad, sexo, IMC y FGe, no puede descartarse confusión residual.

Aspectos éticos. El estudio se realizó conforme a la Declaración de Helsinki y a las recomendaciones del ICMJE. El comité de ética de la investigación con medicamentos correspondiente aprobó el protocolo y, dada la naturaleza retrospectiva del estudio, dispensó el consentimiento informado individual.

## Resultados

### Características de la cohorte

Las características basales y de seguimiento, estratificadas por riesgo de caídas, se presentan en la tabla 1. El CCI mediano fue de 4 (media 5,18; DE 2,31), con distribución equilibrada entre estratos bajo (CCI < 5: 50,3%) y alto (CCI ≥ 5: 49,7%). El UALR basal mostró una distribución asimétrica y leptocúrtica (mediana 2,25; asimetría 2,75; curtosis 13,94) y aumentó una media de 0,34 unidades (13,5%) a los 5 años. Los participantes con riesgo de caídas presentaron mayor CCI (6,53 frente a 4,95; p < 0,001), mayor UALR basal (2,85 ± 1,54 frente a 2,46 ± 1,34) y menor recuento linfocitario basal (2,22 ± 0,80 frente a 2,46 ± 0,81).

**Tabla 1:** Características de la cohorte estratificadas por riesgo de caídas incidente (Downton ≥ 3) durante el seguimiento (n = 310)

Variable	Total (n = 310)	Sin riesgo (n = 265)	Con riesgo (n = 45)	p
Edad (años), media	76,4	76,0	78,4	0,001
Sexo femenino, n (%)	163 (52,6)	143 (54,0)	20 (44,4)	0,237
CCI, media ± DE	5,18 ± 2,31	4,95 ± 2,18	6,53 ± 2,60	< 0,001
CCI ≥ 5, n (%)	154 (49,7)	122 (46,0)	32 (71,1)	0,003
IMC, media ± DE	28,6 ± 3,4	28,5 ± 2,8	28,8 ± 5,5	0,725
Tiazidas, n (%)	31 (10,0)	27 (10,2)	4 (8,9)	0,788
UALR basal, media ± DE	2,52 ± 1,37	2,46 ± 1,34	2,85 ± 1,54	0,080
UALR final, media ± DE	2,86 ± 1,71	2,71 ± 1,60	3,71 ± 2,06	0,003
ΔUALR, media ± DE	0,34 ± 1,55	0,25 ± 1,47	0,86 ± 1,90	0,043
Ácido úrico basal, mg/dl	5,34 ± 1,72	5,32 ± 1,45	5,50 ± 1,73	0,521
Linfocitos basal, × 10^9^/l	2,42 ± 0,81	2,46 ± 0,81	2,22 ± 0,80	0,064

El UALR basal presentó asimetría 2,75 y curtosis 13,94. Comparaciones: variables continuas, t de Student o U de Mann-Whitney; categóricas, χ^2^ o test exacto de Fisher.

CCI: índice de comorbilidad de Charlson; DE: desviación estándar; IMC: índice de masa corporal; UALR: cociente ácido úrico/linfocitos; ΔUALR: cambio del UALR (final − basal).

### Medición principal: CCI → UALR → riesgo de caídas

El efecto indirecto a través del UALR representó aproximadamente un tercio de la asociación entre el CCI y el riesgo de caídas incidente (tabla 2) (fig. 2). La vía a (CCI → UALR) fue significativa (B = 0,099; EE = 0,034; p = 0,003) y la vía b (UALR → riesgo de caídas), de mayor magnitud (B = 0,402; EE = 0,100; p < 0,001; OR = 1,49), unas 4 veces superior. El efecto indirecto fue significativo (a × b = 0,040; IC del 95% BCa 0,007-0,072; p = 0,016) y representó el 33,8% del efecto total; no obstante, la proporción mediada no alcanzó la significación (p = 0,054; IC del 95%, 0,006-0,682), por lo que el mensaje se sustenta en el efecto indirecto y no en dicha proporción. El efecto directo quedó en significación limítrofe (c’ = 0,078; p = 0,060). El modelo explicó el 16,2% de la varianza del desenlace en la escala latente.

**Tabla 2:** Modelo de mediación comorbilidad → UALR → riesgo de caídas (SEM-WLSMV; n = 310)

Vías / efectos	B	EE	z	p	β
*Coeficientes de las vías*					
Vía a (CCI → UALR)	0,099	0,034	2,938	0,003	0,222
Vía b (UALR → riesgo de caídas)	0,402	0,100	4,023	< 0,001	0,381
Efecto directo c’	0,078	0,041	1,884	0,060	0,165
*Covariables*					
Edad → UALR	0,004	0,018	0,223	0,823	0,017
Sexo → UALR	0,058	0,158	0,366	0,715	0,028
Edad → riesgo de caídas	0,050	0,021	2,392	0,017	0,202
Sexo → riesgo de caídas	−0,223	0,190	−1,174	0,240	−0,103
*Efectos de mediación*					
Indirecto (a × b)	0,040	0,016	2,414	0,016	—
Total (c’ + a × b)	0,118	0,044	2,660	0,002	—
Proporción mediada (33,8%)	0,338	0,176	1,928	0,054	—

Estimador WLSMV; IC de la mediación mediante bootstrap BCa (5.000 remuestreos). R^2^ = 0,162. El efecto indirecto fue significativo (IC 95%: 0,007-0,072); la proporción mediada no (IC del 95%, 0,006-0,682).

B: coeficiente no estandarizado; β: coeficiente estandarizado; CCI: índice de comorbilidad de Charlson;; EE: error estándar robusto.

**Figura 2:**
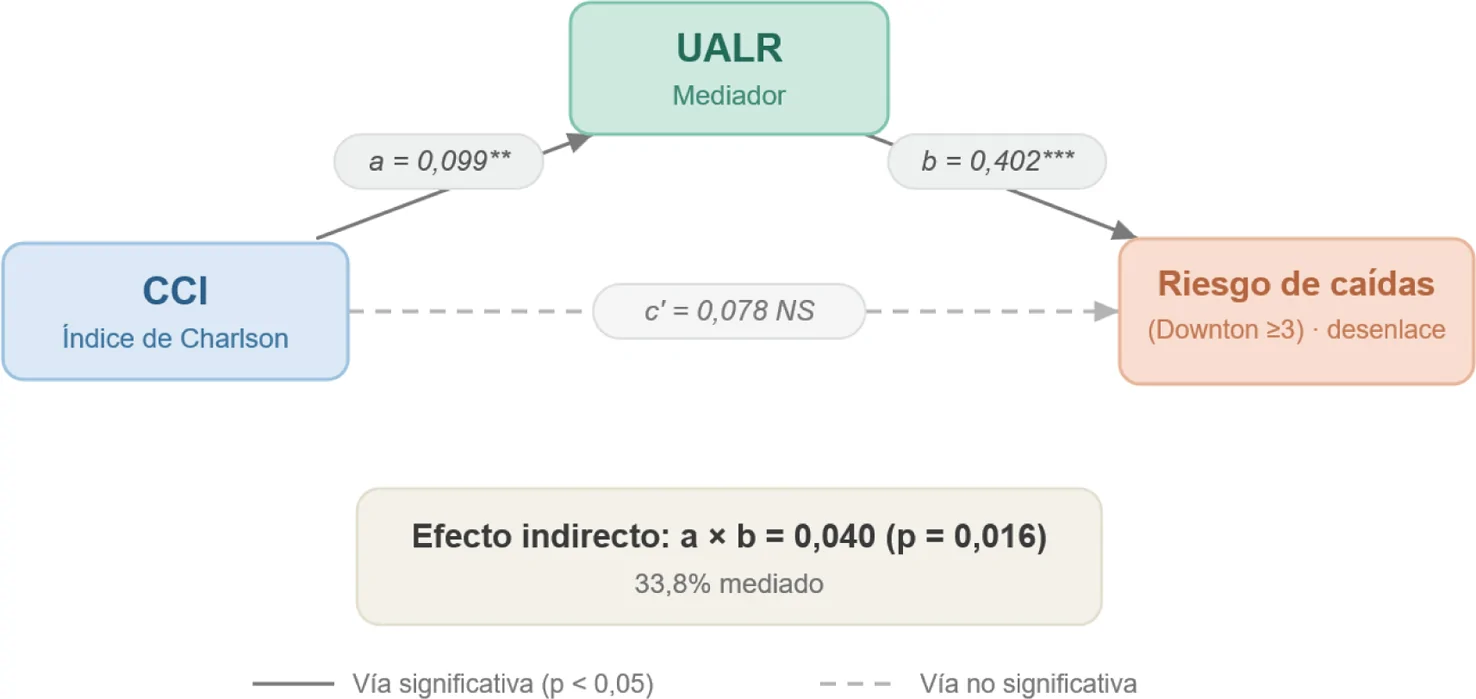
Modelo de mediación de la asociación entre comorbilidad (CCI) y riesgo de caídas (Downton ≥ 3) a través del UALR. Coeficientes no estandarizados (estimador WLSMV). a: vía CCI → UALR; b: vía UALR → riesgo de caídas; c’: efecto directo. Efecto indirecto a × b = 0,040 (IC del 95%, 0,007-0,072; p = 0,016). Líneas continuas: vías significativas; discontinuas: no significativas.

### Dimensión temporal y evolución del UALR

Al sustituir el UALR basal por su cambio (tabla 3), las 3 vías se mantuvieron significativas (vía a: B = 0,111; p < 0,001; vía b: B = 0,135; p < 0,001; vía c’: B = 0,102; p = 0,009), pero el efecto indirecto descendió (a × b = 0,015; p = 0,014; 12,8% mediado), lo que sugiere que el UALR basal es mejor mediador que su incremento. En el modelo mixto, el UALR aumentó durante el seguimiento (p < 0,001), con el sexo masculino y el CCI como predictores independientes; el ajuste por FGe apenas modificó el coeficiente (−0,5%). El coeficiente de correlación intraclase (0,481) indicó que casi la mitad de la varianza del UALR correspondía a diferencias estables entre sujetos.

**Tabla 3:** Modelo de mediación con cambio longitudinal: comorbilidad → ΔUALR → riesgo de caídas (SEM-WLSMV; n = 310)

Vías / efectos	B	EE	z	p	β
*Coeficientes de las vías*					
Vía a (CCI → ΔUALR)	0,111	0,032	3,466	< 0,001	0,166
Vía b (ΔUALR → riesgo de caídas)	0,135	0,038	3,545	< 0,001	0,193
Efecto directo c’	0,102	0,039	2,601	0,009	0,218
*Efectos de mediación*					
Indirecto (a × b)	0,015	0,006	2,457	0,014	—
Total (c’+ a × b)	0,117	0,038	3,059	0,002	—
Proporción mediada (12,8%)	0,128	0,070	1,844	0,065	—

ΔUALR: cambio a 5 años del UALR. Estimador WLSMV; IC mediante bootstrap BCa (5.000 remuestreos).

B: coeficiente no estandarizado; β: coeficiente estandarizado; CCI: índice de comorbilidad de Charlson; EE: error estándar robusto.

### Invarianza por sexo

En el modelo multigrupo no restringido, en mujeres fueron significativas la vía a (B = 0,135; p = 0,013) y la vía b (B = 0,446; p = 0,001), mientras que en varones solo la vía b (B = 0,353; p = 0,016). El contraste de invarianza no detectó diferencias significativas (tabla 4; Δχ^2^ = 1,133; gl = 3; p = 0,769), aunque con potencia limitada (20 eventos en mujeres y 25 en varones), por lo que debe interpretarse como ausencia de evidencia de diferencias y no como prueba de equivalencia.

**Tabla 4:** Análisis multigrupo por sexo y contraste de invarianza de Satorra-Bentler (n = 310)

Vía	B	EE	p	β
*Modelo no restringido. Varones*				
CCI → UALR (a)	0,068	0,045	0,131	0,163
UALR → riesgo de caídas (b)	0,353	0,146	0,016	0,341
CCI → riesgo de caídas (c’)	0,086	0,053	0,104	0,200
*Modelo no restringido. Mujeres*				
CCI → UALR (a)	0,135	0,054	0,013	0,276
UALR → riesgo de caídas (b)	0,446	0,136	0,001	0,420
CCI → riesgo de caídas (c’)	0,057	0,067	0,393	0,110
*Modelo restringido (vías iguales entre sexos)*				
CCI → UALR (a)	0,095	0,035	0,006	0,226/0,199
UALR → riesgo de caídas (b)	0,403	0,100	< 0,001	0,393/0,373
CCI → riesgo de caídas (c’)	0,075	0,042	0,072	0,173/0,145
*Contraste de invarianza (Satorra-Bentler)*				
χ^2^ = 1,133; gl = 3; p = 0,769 — sin diferencias significativas (potencia limitada)				

Estimador WLSMV. Eventos: 20 en mujeres, 25 en varones.

B: coeficiente no estandarizado; β: estandarizado (varones/mujeres en el modelo restringido); EE: error estándar robusto.

#### Análisis de sensibilidad y exploratorios

El efecto mediador del UALR basal fue concordante entre métodos y plataformas (material suplementario, tabla S1): mediante SEM-WLSMV con bootstrap BCa (a × b = 0,040) y mediante mediación simple con desenlace logístico (PROCESS, modelo 4, en SPSS, replicado en R) con bootstrap de percentiles (a×b = 0,038; IC del 95% 0,002-0,085), ambos significativos. En cambio, la mediación del ΔUALR fue significativa en el SEM (a × b = 0,015; p = 0,014) pero no en PROCESS (a × b = 0,021; IC del 95% −0,006-0,060), en coherencia con la significación marginal del path b (p = 0,060) y el distinto marco de estimación (probit latente frente a logit observado); ello confirma que el UALR basal es un mediador más robusto que su cambio. En los modelos de mediación serial (anexo, material suplementario, figura S1), el UALR se asoció con cambios marcados en los biomarcadores distales (vía d_21_ significativa para PLR, ácido úrico, albúmina y NLR; todos p ≤ 0,001), pero ninguno de estos se asoció de forma independiente con el desenlace (vía b_2_, todas p > 0,05) y ningún efecto indirecto serial alcanzó significación.

## Discusión

El efecto indirecto a través del UALR representó aproximadamente un tercio de la asociación total observada entre la comorbilidad y el riesgo de caídas, de forma consistente entre métodos y sin diferencias significativas por sexo. La atenuación del efecto directo hasta una significación limítrofe no debe interpretarse como mediación completa, dado que la significación de c’ depende del tamaño muestral. El predominio de la vía b sobre la vía a es compatible con que el UALR, una vez elevado, se asocie con el riesgo de caídas con más fuerza que la propia comorbilidad, aunque el carácter observacional del estudio impide atribuir causalidad.

### Validez de constructo y naturaleza del desenlace

El índice de Downton mide riesgo de caídas y no fragilidad; su uso como indicador de vulnerabilidad funcional se apoya en el solapamiento con el fenotipo de Fried y en su asociación con la fragilidad^8^, pero no equivale a un diagnóstico con instrumentos validados. Por otra parte, la disponibilidad de la puntuación de Downton en la evaluación basal, sin ningún caso con riesgo elevado al inicio, permite interpretar los 45 casos como incidentes y establece la temporalidad entre los biomarcadores basales y el desenlace, lo que refuerza la plausibilidad del papel mediador del UALR.

### Comparación con la literatura y plausibilidad biológica

Frente a la PCR o la IL-6, con capacidad predictiva longitudinal limitada^11^, el UALR mostró una asociación significativa con el riesgo de caídas; su naturaleza integradora podría captar dimensiones complementarias^13^, si bien la comparación con biomarcadores combinados (que explican un 10-12% de la varianza^12^) es indirecta. La plausibilidad biológica se apoya en la generación de especies reactivas de oxígeno por la xantina oxidasa^14^, ^15^, que favorecen disfunción endotelial y sarcopenia^28^, y en el declive linfocitario por inmunosenescencia^16^ y activación neurohumoral^29^, en línea con la mediación descrita entre el ácido úrico y los eventos cardiovasculares a través del NLR^30^. Pese al dimorfismo del ácido úrico^20^, la mediación fue invariante por sexo.

### Limitaciones

Este estudio tiene varias limitaciones. Su carácter exploratorio y el reducido número de eventos (45 caídas) limitan la potencia, favorecen el sobreajuste y obligan a interpretar los modelos multigrupo y seriales, y la proporción mediada (no significativa), como generadores de hipótesis. El elevado porcentaje de exclusiones (820/1.130; 72,6%), en su mayoría por datos de laboratorio incompletos, y el carácter de análisis secundario de una cohorte no diseñada para esta pregunta introducen un posible sesgo de selección: es probable que se seleccionaran pacientes con mayor contacto con el sistema sanitario y mejor situación funcional, lo que podría atenuar las asociaciones observadas. Además, la realización en un único centro de salud, la inclusión exclusiva de pacientes con seguimiento activo, la restricción a personas de 70-85 años no institucionalizadas y clínicamente estables y la elevada proporción de exclusiones restringen la extrapolación a la población general. El diseño observacional y el supuesto no verificable de ausencia de confusión no medida restringen la inferencia causal; asimismo, no se dispuso de otros factores bien conocidos del riesgo de caídas —polifarmacia, deterioro cognitivo, dependencia funcional, antecedentes de caídas, osteoporosis, uso de benzodiazepinas o estado nutricional—, cuya ausencia podría haber influido en la magnitud del efecto estimado. Finalmente, la disponibilidad de solo 2 mediciones impide modelar trayectorias, y la ausencia de PCR e IL-6 impidió la comparación directa con biomarcadores convencionales.

### Aplicabilidad y líneas futuras

El UALR se obtiene sin coste adicional de una analítica rutinaria. Con carácter exploratorio, cabe plantear la hipótesis —no una conclusión de este estudio— de que pudiera aportar información de estratificación complementaria a la comorbilidad; su utilidad clínica real es desconocida y debería evaluarse en estudios diseñados a tal efecto. Antes de cualquier uso clínico, estos hallazgos deben validarse en cohortes prospectivas independientes con instrumentos de fragilidad estandarizados, compararse con biomarcadores inflamatorios convencionales y evaluarse si intervenciones sobre el ácido úrico o la reserva inmunitaria modifican el riesgo.

## Conclusiones

En esta cohorte, el efecto indirecto a través del UALR en la asociación entre la carga de comorbilidad y el riesgo de caídas incidente fue estadísticamente significativo, sin evidencia de diferencias por sexo. No obstante, dado su carácter exploratorio y el reducido número de eventos (45), estos resultados deben interpretarse como generadores de hipótesis. El UALR es un índice obtenible sin coste de una analítica rutinaria, y la hipótesis de su posible utilidad como marcador complementario de estratificación debería confirmarse en cohortes prospectivas independientes antes de cualquier aplicación clínica.

## Contribución de los autores

AAT: conceptualización, metodología, análisis formal, redacción del borrador original. ASJ: análisis de datos, revisión y edición. JAG: conceptualización, metodología, supervisión, revisión y edición. Todos los autores tuvieron acceso a los datos y aprobaron la versión final.

## Consideraciones éticas

El estudio se realizó conforme a la Declaración de Helsinki y a las recomendaciones del ICMJE. El proyecto (código CHUNSC_2025_04) fue aprobado por el Comité de Ética de la Investigación con medicamentos (CEIm) provincial de Santa Cruz de Tenerife, Complejo Hospitalario Universitario de Canarias.

## Declaración de la IA generativa y las tecnologías asistidas por IA en el proceso de escritura

Durante la preparación de este trabajo los autores utilizaron una herramienta de IA generativa con el fin de mejorar la legibilidad y el estilo lingüístico del texto. Tras utilizar dicha herramienta, los autores revisaron y editaron el contenido según fue necesario y asumen la plena responsabilidad del contenido de la publicación.

## Financiación

Esta investigación no recibió ninguna ayuda específica de organismos públicos, comerciales o sin ánimo de lucro.

**Lo conocido sobre el tema**

– La fragilidad y la comorbilidad aumentan el riesgo de caídas en los mayores, pero los biomarcadores inflamatorios unidimensionales (PCR, IL-6) tienen escasa capacidad predictiva longitudinal.
– El cociente ácido úrico/linfocitos (UALR) integra estrés oxidativo e inmunosenescencia y se ha asociado a peor pronóstico en contextos clínicos agudos.
– Se desconocía su papel en población comunitaria y su relación con el riesgo de caídas.

**Qué aporta este estudio**

– El efecto indirecto a través del UALR representó aproximadamente un tercio de la asociación entre comorbilidad y riesgo de caídas incidente, de forma consistente entre métodos.
– Su asociación con el riesgo de caídas es mayor que la de la propia comorbilidad y no difiere por sexo.
– Es un índice obtenible sin coste de una analítica rutinaria, con potencial para la estratificación del riesgo en Atención Primaria.

## Conflicto de intereses

Los autores declaran no tener conflictos de interés.

## Disponibilidad de datos

Los datos están disponibles previa solicitud al autor de correspondencia.
