# Supplementary material for: Uric acid-to-lymphocyte ratio as a mediator between comorbidity and fall risk in older adults
Source: Aten Primaria. 2026 Sep 22;58(12):103611. [Article in Spanish] doi: 10.1016/j.aprim.2026.103611 (PMC13633936; doi:10.1016/j.aprim.2026.103611)
Supplement: Supplementary file 1 [file mmc1.doc]

**Material Suplementario**

**Tabla S1. Evidencia convergente de la mediación a través del UALR mediante tres métodos analíticos (n=310).**

| **Método** | **Mediador** | **Efecto indirecto** | **% mediado** | **Significativo** |
| --- | --- | --- | --- | --- |
| SEM-WLSMV | UALR basal | 0,040 (p=0,016) | 33,8 | Sí |
| PROCESS M4 (SPSS) | UALR basal | 0,038 (IC 0,002-0,085) | — | Sí |
| Bootstrap BCa | UALR basal | 0,040 (IC 0,007-0,072) | 33,8 | Sí |
| SEM-WLSMV | ΔUALR | 0,015 (p=0,014) | 12,8 | Sí |
| PROCESS M4 (SPSS) | ΔUALR | 0,021 (IC −0,006-0,060) | — | No |

*IC: intervalo de confianza al 95% (bootstrap BCa en lavaan; bootstrap de percentiles en PROCESS). El UALR basal fue mediador significativo en ambos métodos; la mediación del ΔUALR solo alcanzó significación en el SEM. La mediación con UALR basal fue 2,6 veces mayor que con el ΔUALR; Efectos de PROCESS en escala log-odds. la discrepancia de PROCESS frente a SEM se atribuye al distinto estimador.*

**Figura S1.** *Modelos de mediación serial del efecto de la comorbilidad sobre el riesgo de caídas a través del UALR y de biomarcadores distales (PLR, NLR, albúmina, ácido úrico).* ***CCI → UALR → M2 → riesgo de caídas.*** *M2: segundo mediador en serie.* *Se muestran los coeficientes no estandarizados (B). Las flechas continuas representan trayectorias significativas (p < 0,05), mientras que las flechas discontinuas representan trayectorias no significativas. Ningún efecto indirecto serial (a₁ × d₂₁ × b₂) alcanzó significación (todos p > 0,35), lo que indica que ninguno de los segundos mediadores (PLR, NLR, albúmina o AU) transmite el efecto del CCI sobre el riesgo de caídas a través de la secuencia UALR → M2.*

**Lista de comprobación STROBE (estudios de cohortes)**

| **Nº** | **Recomendación STROBE (estudios de cohortes)** | **Ubicación en el manuscrito** |
| --- | --- | --- |
| **Título y resumen** |  |  |
| 1a | Diseño del estudio indicado en título o resumen | Título; Resumen (Diseño) |
| 1b | Resumen informativo y equilibrado | Resumen estructurado |
| **Introducción** |  |  |
| 2 | Antecedentes y justificación científica | Introducción |
| 3 | Objetivos e hipótesis específicas | Introducción (último párrafo) |
| **Métodos** |  |  |
| 4 | Elementos clave del diseño del estudio | Material y métodos: Diseño, población y selección |
| 5 | Contexto, lugares y fechas relevantes (2019-2024) | Diseño, población y selección |
| 6 | Criterios de elegibilidad, fuentes y métodos de selección de participantes | Diseño, población y selección; Figura 1 (esquema del estudio) |
| 7 | Variables: desenlace, exposición, mediador y covariables | Variables |
| 8 | Fuentes de datos y métodos de medida (historia clínica electrónica; analítica) | Variables |
| 9 | Medidas para afrontar posibles fuentes de sesgo | Análisis estadístico; Discusión (Limitaciones) |
| 10 | Justificación del tamaño muestral (eventos por parámetro) | Análisis estadístico: Consideraciones sobre el tamaño muestral |
| 11 | Tratamiento de las variables cuantitativas | Variables; Análisis estadístico |
| 12 | Métodos estadísticos (SEM-WLSMV, mediación, invarianza; R y SPSS-PROCESS) | Análisis estadístico |
| **Resultados** |  |  |
| 13 | Participantes: flujo, elegibles, incluidos y exclusiones | Figura 1; Resultados (Características de la cohorte) |
| 14 | Datos descriptivos de los participantes y covariables | Tabla 1; Resultados (Características de la cohorte) |
| 15 | Datos del desenlace (nº de eventos; desenlace incidente) | Resultados; Tabla 1 |
| 16 | Resultados principales: estimaciones ajustadas e IC | Tablas 2-4; Resultados |
| 17 | Otros análisis (sensibilidad, exploratorios, convergencia entre métodos) | Resultados (Análisis de sensibilidad y exploratorios); material suplementario (Tabla S1, Figura S1) |
| **Discusión** |  |  |
| 18 | Resultados clave en relación con los objetivos | Discusión (primer párrafo) |
| 19 | Limitaciones (sesgo de selección, potencia, imprecisión) | Discusión (Limitaciones) |
| 20 | Interpretación global y cautelosa | Discusión (Validez de constructo y naturaleza del desenlace; Comparación con la literatura y plausibilidad biológica) |
| 21 | Generalización (validez externa) | Discusión (Limitaciones; Aplicabilidad y líneas futuras) |
| **Otra información** |  |  |
| 22 | Financiación y papel de los financiadores | Página de título (Financiación) |

*Adaptado de von Elm E, et al. Declaración STROBE (www.strobe-statement.org). El estudio es un análisis secundario de una cohorte retrospectiva; el desenlace (riesgo de caídas, índice de Downton ≥3) se registró en la evaluación basal y final, sin casos prevalentes en la basal.*
